# pH- and concentration-dependent supramolecular self-assembly of a naturally occurring octapeptide

**DOI:** 10.3762/bjoc.16.168

**Published:** 2020-08-17

**Authors:** Goutam Ghosh, Gustavo Fernández

**Affiliations:** 1 Organisch-Chemisches Institut, Westfälische Wilhelms-Universität Münster, Correnstraße 40, 48149 Münster, Germany

**Keywords:** aqueous self-assembly, pH-responsive systems, secondary structure, self-assembled nanostructures, solid-phase peptide synthesis

## Abstract

Peptide-based biopolymers represent highly promising biocompatible materials with multiple applications, such as tailored drug delivery, tissue engineering and regeneration, and as stimuli-responsive materials. Herein, we report the pH- and concentration-dependent self-assembly and conformational transformation of the newly synthesized octapeptide PEP-1. At pH 7.4, PEP-1 forms β-sheet-rich secondary structures into fractal-like morphologies, as verified by circular dichroism (CD), Fourier-transform infrared (FTIR) spectroscopy, thioflavin T (ThT) fluorescence spectroscopy assay, and atomic force microscopy (AFM). Upon changing the pH value (using pH 5.5 and 13.0), PEP-1 forms different types of secondary structures and resulting morphologies due to electrostatic repulsion between charged amino acids. PEP-1 can also form helical or random-coil secondary structures at a relatively low concentration. The obtained pH-sensitive self-assembly behavior of the target octapeptide is expected to contribute to the development of novel drug nanocarrier assemblies.

## Introduction

**Scheme 1:**
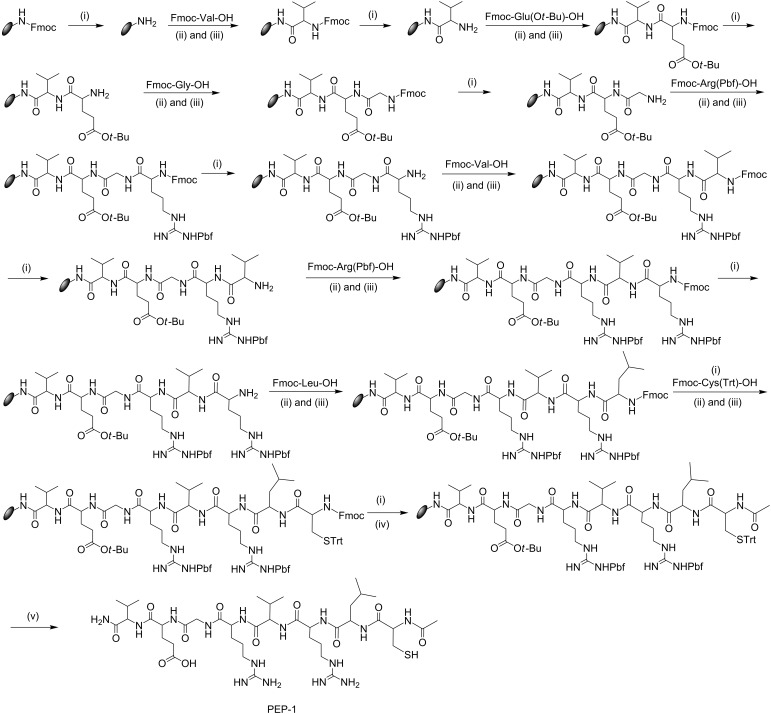
Detailed synthetic scheme for PEP-1. (i) 20% piperidine in DMF, (ii) HBTU, (iii) NMM, (iv) Ac_2_O/Py/DMF 1:2:3 and (v) TFA/phenol/water/TIPS 88:5:5:2.

The self-assembly of small molecules is a ubiquitous phenomenon in nature [1] and also has key implications for the development of a wide range of functional materials [2–8]. Peptides are arguably the most eminent candidates among all types of biological self-assembling building blocks because of a number of key properties. This includes a high biocompatibility [9–14], the ease of materials preparation, and the versatility in tuning their secondary structures (e.g., β-sheet and α-helix) by designing the amino acid sequence [15]. Peptidomimetic foldamers designed from nonnatural amino acid sequences are also well characterized systems due to their propensity to form secondary structures, which lead to nanostructured materials [16–17]. Noncovalent interactions, such as hydrogen bonding, van der Waals interactions, hydrophobic interactions, electrostatic interactions, and π–π interactions [18–22] are common driving forces in peptide self-assembly. These noncovalent interactions can also be designed to be responsive to various external stimuli, such as heat, pH, light, enzymes, metal ions, and chemical triggers [23–39]. In this regard, a particularly relevant property of peptide assemblies is their ability to undergo significant changes in their morphologies and secondary structures in response to pH stimuli. For instance, hydrogen bonding interactions are strongly influenced by the pH value, leading to a collapse of the supramolecular aggregates when an acid or base is added [4]. The pH-responsiveness of self-assembled peptides has been extensively exploited for potential applications, with particular focus on drug delivery systems (DDS) [40], as injectable gels for tissue engineering [41–42], and biosensing applications [43]. pH-responsive DDS can deliver the drug to a specific tissue or organ and protect the payload during the passage through physiological barriers. Most importantly, pH-sensitive DDS are considered as suitable carriers for chemotherapeutics [44–46]. Furthermore, peptides also play an important role as active moieties for many diseases, including cancer [47], peptic ulcer [48], asthma [49], cardiovascular diseases [50], and hypertension [51]. A particularly important aspect of peptides that contain both hydrophobic and hydrophilic amino acid residues is their amphiphilicity, which plays a crucial role in the self-assembly process [52]. Keeping these factors in mind, we synthesized an octapeptide, PEP-1, which contains two valine (Val) units and one leucine (Leu) unit as the hydrophobic residues and one glutamic acid (Glu) residue, two arginine (Arg) units, and one cysteine (Cys) substituent as polar moieties (Scheme 1). This choice of amino acids renders PEP-1 amphiphilic in nature, which is expected to be beneficial for aqueous self-assembly. The polar Glu and Arg motifs are also sensitive towards basic and acidic pH values, respectively. PEP-1 is actually a naturally occurring β-strand peptide fragment (residues 16−23 in Figure S1, Supporting Information File 1) of galectin-1, a β-sheet lectin protein that is available in bovine spleen [53] (for a detailed crystal structure see the Protein Data Bank; PDB ID 1SLT). Given the amphiphilic and pH-responsive nature of PEP-1, we investigated both the pH- and concentration-dependent formation of nanostructures as well as the secondary structures.

## Results and Discussion

### Solid-phase peptide synthesis and purification

The target octapeptide was synthesized in the solid phase following four steps, including: i) deprotection of the Fmoc protecting group, ii) coupling of an amino acid, iii) cleavage of the peptide from the solid support, and iv) purification of the peptide by reversed-phase HPLC. Fmoc-protected Rink amide resin (0.45 g, 0.5 mmol) was swelled in 10 mL of DMF for two hours. After that, the swelled resin was loaded into the special apparatus [23], and the solvent was drained off. The swelled resin was washed with DMF (10 mL × 4). Subsequently, 10 mL of 20% piperidine in DMF were added to the preswollen resin, and the resulting mixture was stirred for 30 minutes under a nitrogen gas atmosphere. After washing the resin with DMF, the deprotection procedure was repeated, and the resin was thoroughly washed with DMF. Subsequently, a Kaiser test [23] was performed to monitor the deprotection step. A few resin beads were placed in a small vial and washed with ethanol, and then, two drops of each of the three solutions were added and heated to 100 °C for 4–6 min. The color change of the initially colorless beads to blue/purple revealed that the test was positive, indicating that the deprotection was complete and that the system was ready for the coupling procedure.

The solution of the Fmoc-protected amino acid (2 mmol, 4 equiv), HBTU (2 mmol, 4 equiv), and NMM (8 mmol, 16 equiv) in 10 mL DMF was added to the resin, and the resulting mixture was allowed to stir for two hours under a nitrogen gas environment. After the completion of the reaction, the solution was drained off, and the resin was washed as mentioned previously (with DMF; 4–6 times for 30 seconds). The completion of the coupling procedure was confirmed by a Kaiser test. Several cycles of deprotection, coupling, and washing procedures were repeated until the desired peptide was obtained. The N-terminus of the peptide was acetylated by adding 10 mL of a mixture of acetic anhydride/pyridine/DMF 1:2:3 to the peptidyl resin at room temperature, and this was stirred for 2 hours following the same procedure as before.

At the final stage, we cleaved the peptide from the resin by using a proper cleavage cocktail: TFA/phenol/water/TIPS 88:5:5:2. DTT was included, as this peptide contains cysteine. After adding the cleavage cocktail to the dried resin and stirring for 2 hours, the solution was drained off and the resin was washed with the cocktail and concentrated to dryness in a round-bottom flask. The peptide was washed several times with cold ether, subsequently dissolved in distilled water or glacial acetic acid, and then lyophilized. The lyophilized peptide was dissolved in water/acetonitrile 1:1, v/v, in the presence of 0.1% TFA and purified by RP-HPLC using eluent A (10% acetonitrile, 90% water containing 0.1% TFA) and eluent B (80% acetonitrile, 20% water containing 0.1% TFA) in a linear acetonitrile→water gradient (11% B→50% B in 40 min at 25 °C ) on a SymmetryPrepTM C18 preparative column (7 µm, 7.8 × 300 mm) at a flow rate of 2 mL/min. Peaks were detected at 214 nm. The desired peak was collected, and the purity was checked in an analytical Symmetry C18 column (5 µm, 4.6 × 250 mm). The single peak demonstrated the purity of the peptide (Figure S2, Supporting Information File 1). The identity of the peptide was confirmed by MALDI–TOF mass spectrometry (Figure S3, Supporting Information File 1). The yield of the purified PEP-1 was 42%.

### Self-assembly and secondary-structure formation

**Figure 1:**
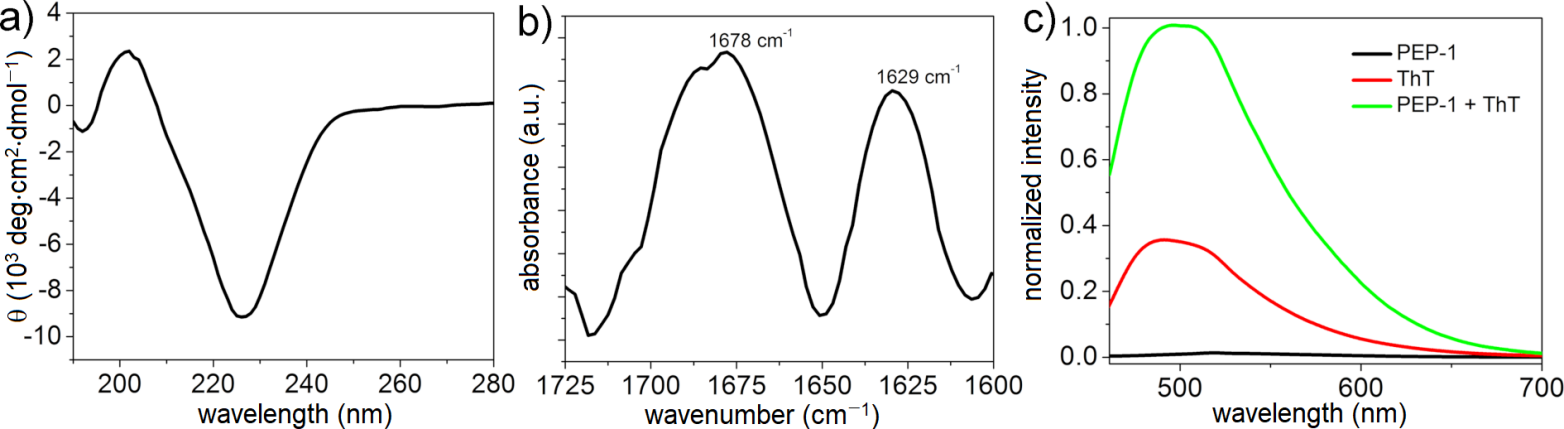
a) CD spectrum; b) FTIR spectrum of the amide I region; and c) ThT fluorescence assay of PEP-1 in PBS at pH 7.4 (*c* = 5.0 × 10^−4^ M).

CD, FTIR spectroscopy, and ThT fluorescence spectroscopy assay were used to investigate the formation of secondary structures from PEP-1 during the self-assembly. The CD spectrum of PEP-1 at pH 7.4 (PBS buffer, *c* = 5.0 × 10^−4^ M) showed an intense negative band at around 226 nm that indicated the characteristic signature of a β-sheet-rich structure (Figure 1a) [54–56].

To support the results obtained from CD spectroscopy, FTIR spectroscopy was performed in D_2_O (pH 7.4). The appearance of two intense peaks at 1629 and 1678 cm^−1^ in the amide I region (Figure 1b) suggested an intermolecular antiparallel β-sheet arrangement [57–60]. The band at 1678 cm^−1^ was the characteristic feature of an antiparallel conformation of the sheet structure or the β-turn structure [61]. To further confirm the β-sheet formation, we performed a ThT fluorescence spectroscopy assay. ThT is a widely used fluorescent dye that is amyloid-specific and can bind specifically to multistranded β-sheets [62–63]. PEP-1 is nonemissive due to the absence of chromophores in its molecular structure, whereas ThT shows a low fluorescence in PBS (pH 7.4) upon excitation at 440 nm. Interestingly, the fluorescence intensity of ThT significantly increases upon mixing with PEP-1 (Figure 1c), confirming the formation of a β-sheet structure and supporting the results obtained from CD and FTIR spectroscopy.

**Figure 2:**
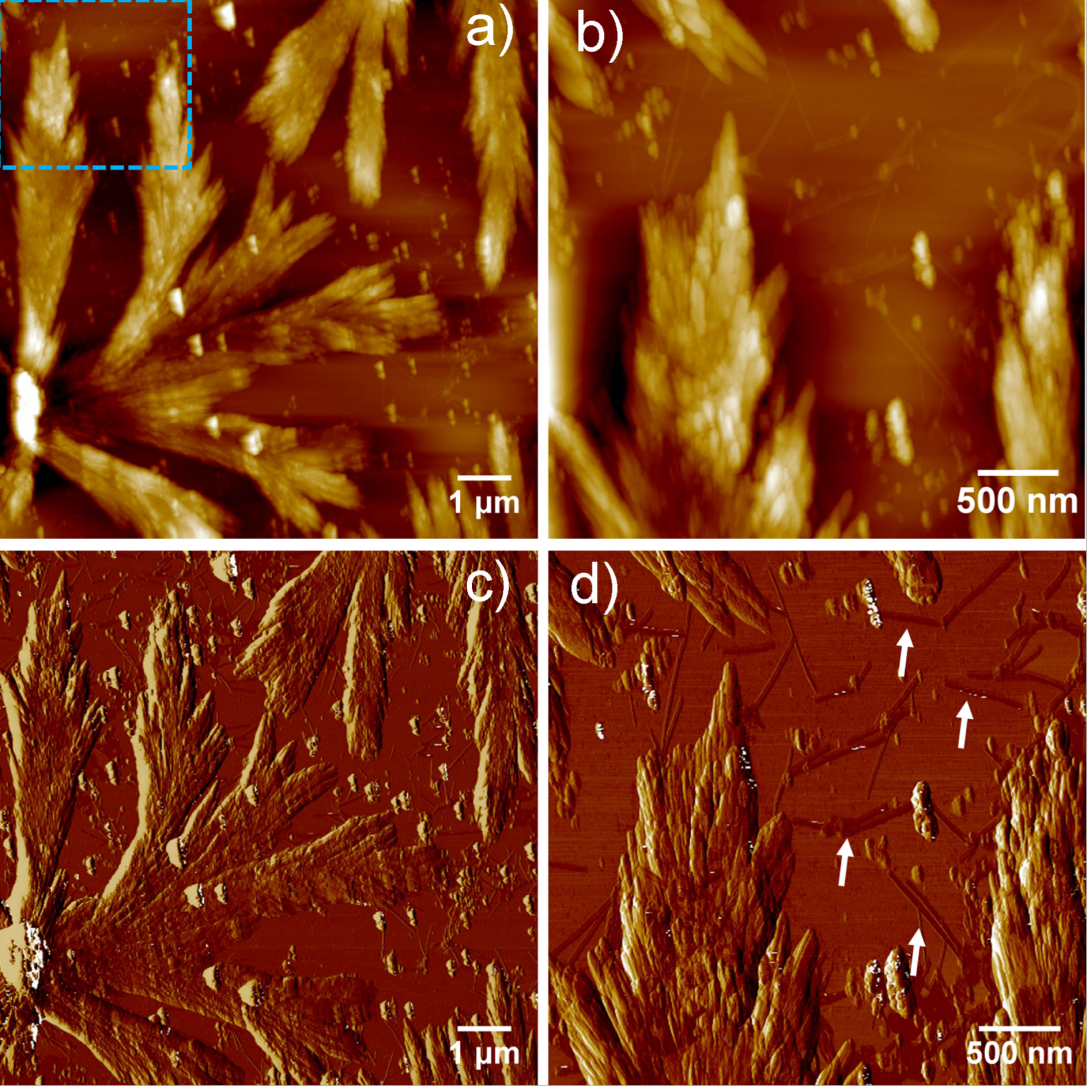
AFM height (a and b) and corresponding phase images (c and d) of PEP-1 at pH 7.4 on mica (*c* = 5.0 × 10^−4^ M).

Microscopic studies by AFM revealed the formation of fractal-like structures (Figure 2a and Figure 2c) of several micrometers in length along with discrete short and rigid nanobelts, as evident from the zoomed height and phase images (Figure 2b and Figure 2d). These results imply that PEP-1 first self-assembles into nanobelts, which further assemble into larger structures in a hierarchical process [64].

This behavior may be the result of strong electrostatic attraction forces between the peptide molecules at a neutral pH value. Even though the pH-responsive behavior of peptides is a well-known phenomenon, fractal-like-structure formation from β-sheets has been rarely observed previously [65].

### Effect of pH on the self-assembly

**Figure 3:**
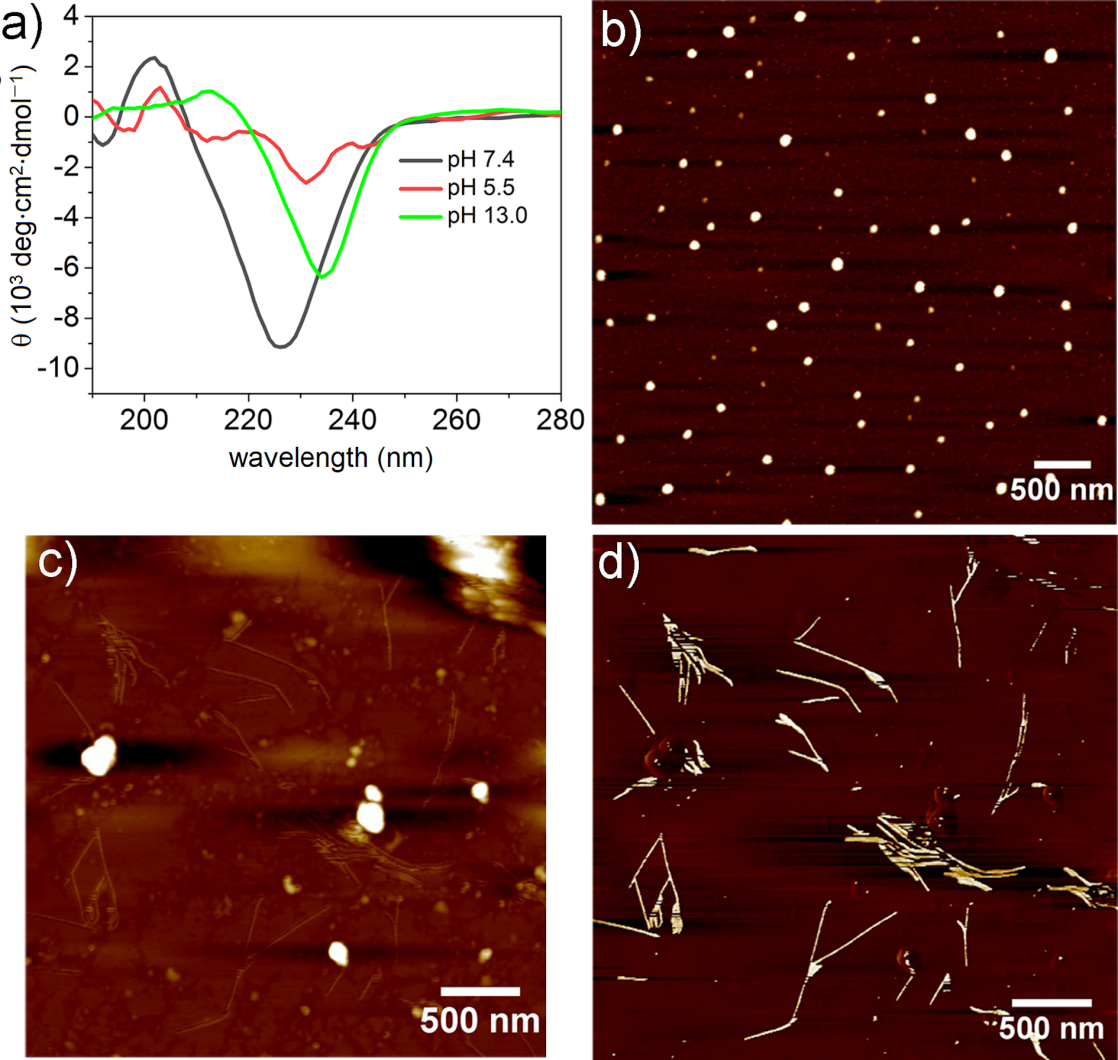
a) pH-dependent CD spectra of PEP-1. b) AFM height image at pH 5.5; c) at pH 13.0; and d) corresponding phase image of c) on mica (*c* = 5.0 × 10^−4^ M).

The presence of pH-responsive amino acids, such as two Arg (containing free amine groups) and two Glu residues (containing free acids) prompted us to investigate the effect of the pH value on the self-assembly and secondary structures. For this, we performed CD and AFM (Figure 3 and Figure S4, Supporting Information File 1) both in acidic (pH 5.5 and 2.2) and basic (pH 13.0 and 10.3) media. The pH-dependent CD spectra of PEP-1 revealed that the β-sheet secondary structure completely dissociates upon altering the pH from 7.4 to 5.5 (Figure 3a, red spectrum), which can be explained by strong electrostatic repulsions involving Arg^+^ moieties. AFM studies at pH 5.5 showed the formation of nanoparticle assemblies, which is in agreement with the loss of the secondary structure (Figure 3b). Similarly, at pH 2.2, PEP-1 also exhibited a structureless CD spectrum and irregular nanostructures (Figure S4, Supporting Information File 1). In contrast, the CD spectrum at highly basic conditions, such as at pH 13.0, showed a negative CD signal that was red-shifted (234 nm) and less intense to that observed at pH 7.4 (226 nm), suggesting the formation of a more twisted and distorted β-sheet arrangement [16,66–68]. As the angle between the two peptides increases in twisted β-sheets, the H-bonding distances increase, weakening the intermolecular forces. At basic pH values, the carboxyl group of Glu is present as a negatively charged carboxylate species, thereby inducing weak electrostatic repulsions between Glu^−^ residues, which may be responsible for the lack of well-defined assemblies. This phenomenon is also supported by AFM imaging (Figure 3c and Figure 3d), where PEP-1 forms short fibrillar nanostructures with lengths of several hundred nanometers at pH 13.0. Interestingly, at a lower basic pH value, such as 10.3, PEP-1 formed an almost similar β-sheet conformation and fractal-like morphology as at pH 7.4. This is evidenced by the negative band at 227 nm in the CD spectrum, and further supported by AFM (Figure S4, Supporting Information File 1). At neutral (pH 7.4) and lower basic (pH 10.3) conditions, the self-assembly occurred due to the favorable strong electrostatic attraction between Glu^−^ and Arg^+^ residues [69–70]. However, moving to more acidic or more basic conditions led to less ordered nanoscale morphologies due to the potential participation of the electrostatic repulsions between some of the amino acid residues, as mentioned above. The pH-responsive self-assembling behavior of peptides has a great importance in drug delivery, and since PEP-1 contains rich cationic residues, such as Arg, this system can be an interesting potential candidate for DDS and as an antibacterial agent [71]. Biocompatibility, drug delivery, and antibacterial studies are underway in our laboratory.

### Concentration-dependent secondary structure formation

**Figure 4:**
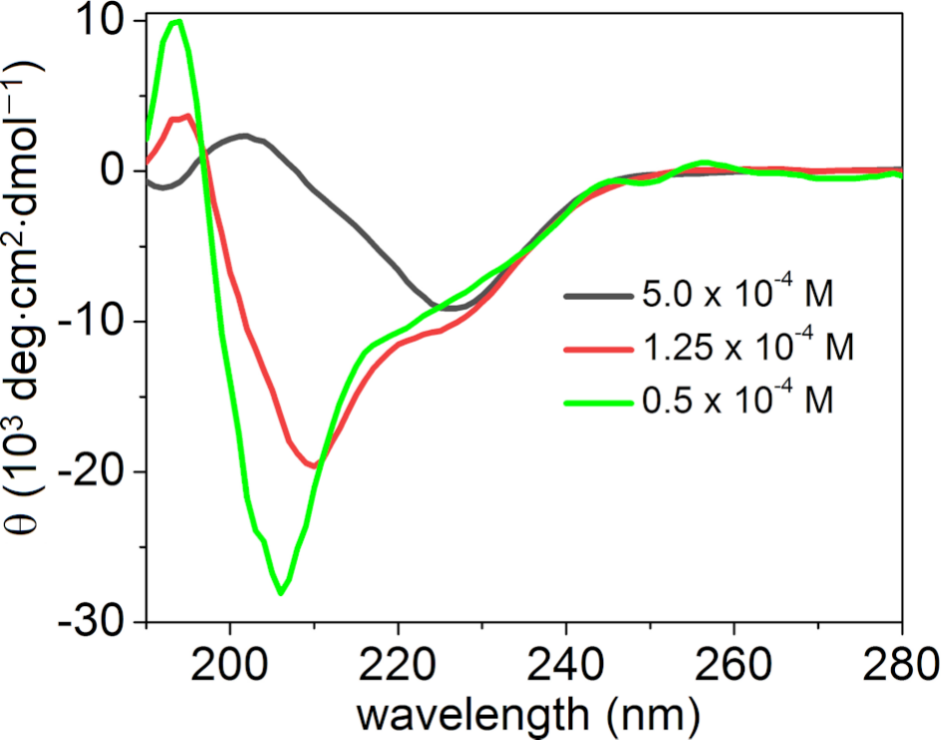
CD spectrum of PEP-1 at different concentrations at pH 7.4.

Apart from the pH-dependent self-assembly, we also investigated the role of the concentration on the self-assembly and secondary structure. As shown previously, the CD spectra at neutral pH disclosed the formation of β-sheet structures at a concentration of 5 × 10^−4^ M in PBS. Intriguingly, lowering the concentration to 1.25 × 10^−4^ M led to a helical secondary structure (Figure 4, red spectrum), which transformed into a random coil structure (Figure 4, green spectrum) upon further decreasing the concentration to 0.5 × 10^−4^ M. To the best of our knowledge, this kind of conformational transformation, dependent on the concentration of the peptide solution, has rarely been reported [72].

We also investigated the time-dependent CD for both the β-sheet (Figure S5, Supporting Information File 1) and the helical conformation (Figure S6, Supporting Information File 1) as well as a ThT assay for the β-sheet (Figure S7, Supporting Information File 1) to better understand a possible conformational transition over time. However, no changes in the particular conformation were found even after 24 h, which suggested the formation of very stable conformations under the investigated conditions. Although the mechanism of transformation is not clear to us at this stage, we hypothesize that multiple intermolecular interactions may play an important role in the transformation. At high concentrations, peptide molecules can come into closer contact and experience strong intermolecular attractive forces to facilitate the β-sheet formation, whereas a low concentration may preferentially favor coiled or helical conformations by salt bridges between positively charged (Arg^+^) and negatively charged (Glu^−^) amino acids [73–74]. Mechanistic insights into the observed conformational transformation via molecular dynamics simulations are underway in our laboratory.

## Conclusion

In summary, we synthesized a naturally occurring amphiphilic peptide fragment, PEP-1, from a β-sheet lectin protein, galectin-1. PEP-1 self-assembles to produce β-sheet-rich structures at physiological pH 7.4, as confirmed by CD, FTIR spectroscopy, and ThT assay. Microscopy studies revealed the hierarchical formation of fractal-like structures from nanobelts. The target peptide PEP-1 appeared to be highly sensitive towards pH changes due to the presence of charged amino acids. Fractal networks and the secondary structure can be dissociated under acidic conditions (pH 5.5) due to strong electrostatic repulsions. Under basic conditions (pH 13.0), the electrostatic repulsions are weakened compared to acidic conditions, but they still have an effect on the secondary structure and the resulting nanoscale morphology, leading to twisted β-sheet structures and short nanofibers. Apart from the pH sensitivity, PEP-1 also showed a concentration dependency of the secondary structure. At high concentration, it formed β-sheet-rich structures, which, upon dilution, transformed into helical structures and further to random coils. Such pH-responsiveness and concentration-dependent conformational changes may provide access to new potential peptide candidates for biomedical applications, which are currently underway in our laboratory.

## Experimental

### FTIR spectroscopy

FTIR spectra were collected on a PerkinElmer Spectrum 100 FTIR spectrometer. A solution of the peptide (*c* = 5.0 × 10^−4^ M) in D_2_O (pH 7.4) was placed in a CaF_2_ cell window with a 0.2 mm spacer. The spectra were recorded against the corresponding solvent background. The scans were between 1800 and 1500 cm^−1^, with 200 accumulations at a resolution of 0.4 cm^−1^.

### CD spectroscopy

CD experiments were performed on a JASCO J-1500 spectropolarimeter equipped with a Peltier module as a temperature controller. The experiments were carried out in buffer solutions at different pH values. The samples for CD were prepared by dissolving the solid peptide in an appropriate buffer solution and measured in the far-UV region at 25 °C in the wavelength range of 280–190 nm. Cuvettes with a path length of 1.0 cm were used. A scan speed of 50 nm/min and a response time of 2.0 s were selected. The spectra were averaged over three scans to minimize signal noise.

### ThT fluorescence spectroscopy assay

For the ThT assay, we used a protocol similar to the one previously described [23]. A diluted solution of ThT at a concentration of 1.0 × 10^−3^ M was prepared in PBS buffer (pH 7.4) from a concentrated stock solution (5.0 × 10^−3^ M) and then filtered with a syringe filter. An aliquot of 50 μL of the diluted ThT solution was added to the peptide solution at 5.0 × 10^−3^ M to reach a final volume of 2000 μL. After that, the mixture was vortexed and kept in the dark for about two hours for binding. After two hours, the fluorescence spectra were registered using an excitation wavelength of 440 nm on a JASCO Spectrofluorometer FP-8500 within a wavelength range between 450 and 700 nm and 5 nm slits.

### AFM studies

For microscopy studies, peptide solutions at a concentration of 5 × 10^−4^ M were prepared at the desired pH values. AFM samples were prepared by drop-casting 10 μL of the respective sample onto a mica surface, followed by spin-coating at 1000 rpm, and then, the thin film was dried at room temperature for 24 h. After that, images were captured on a Multimode^®^ 8 SPM System (AXS Bruker). Silicon cantilevers with a nominal spring constant of 9 Nm^−1^ and with a resonant frequency of ≈150 kHz and a typical tip radius of 7 nm (OMCL-AC200TS, Olympus) were employed.

### Buffer solution preparation

PBS and acetate buffer were used for adjusting pH 7.4 and 5.5, respectively, by following standard protocols. pH 13.0 was prepared by adding NaOH into water. 0.15 M sodium chloride were used to avoid the influence of the ionic strength, and the pH values were precisely measured using a pH meter.

## Supporting Information

File 1 Materials and methods as well as additional figures.
