# Supplementary material for: pH- and concentration-dependent supramolecular self-assembly of a naturally occurring octapeptide
Source: Beilstein J Org Chem. 2020 Aug 17;16:2017–25. doi: 10.3762/bjoc.16.168 (PMC7445398; doi:10.3762/bjoc.16.168)
Supplement: File 1 — Materials and methods as well as additional figures. [file Beilstein_J_Org_Chem-16-2017-s001.pdf]

BEILSTEIN JOURNAL OF ORGANIC CHEMISTRY

# Supporting Information

for

# pH- and concentration-dependent supramolecular self-assembly of a naturally occurring octapeptide

Goutam Ghosh and Gustavo Fernández

*Beilstein J. Org. Chem.* **2020,** *16,* 2017–2025. doi:10.3762/bjoc.16.168

## Materials and methods as well as additional figures

License and Terms: This is a supporting information file under the terms of the Creative Commons Attribution License (http://creativecommons.org/licenses/by/4.0). Please note that the reuse, redistribution and reproduction in particular requires that the authors and source are credited.
The license is subject to the *Beilstein Journal of Organic Chemistry* terms and conditions: (https://www.beilstein-journals.org/bjoc)

## Materials and methods

Rink amide resin (Rink amide 4-methylbenzhydrylamine, polymer-bound), Fmoc-protected amino acids, *O*-(benzotriazol-1-yl)-*N*,*N*,*N*’,*N*’-tetramethyluronium hexafluorophosphate (HBTU), triisopropylsilane (TIPS), trifluoroacetic acid (TFA), and *N*-methylmorpholine (NMM), were purchased from Sigma-Aldrich. Ninhydrin, dimethylformamide (DMF), dichloromethane (DCM), phenol, piperidine, and pyridine were purchased from Merck. All solvents were distilled and dried following standard protocols before use. [1] Reversed-phase high-performance liquid chromatography (RP-HPLC) was done with a Waters HPLC (2489 UV–vis detector) system. An applied Biosystems 4700 Proteomics Analyzer 170 MALDI-TOF mass spectrometer was used to determine the molecular mass of the peptide. Circular dichroism (CD) experiments were performed on a J1500 spectrophotometer from JASCO. FTIR spectra were recorded by using a PerkinElmer Spectrum 100FTIR spectrometer.

## Additional figures

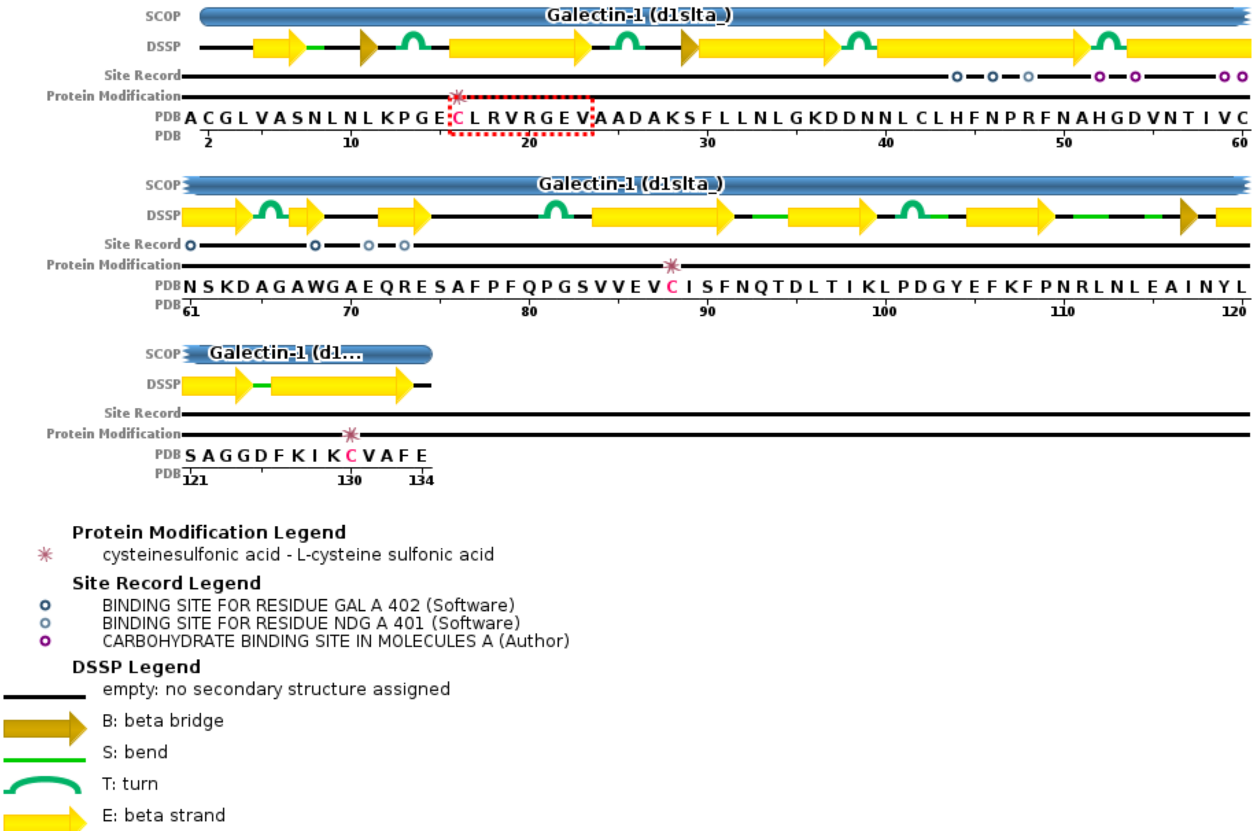


**Figure S1:** Amino acid sequence of galectin-1 from the crystal structure (the red dotted box indicates the PEP-1 sequence). Image from the RCSB PDB (rcsb.org); PDB ID: 1SLT.

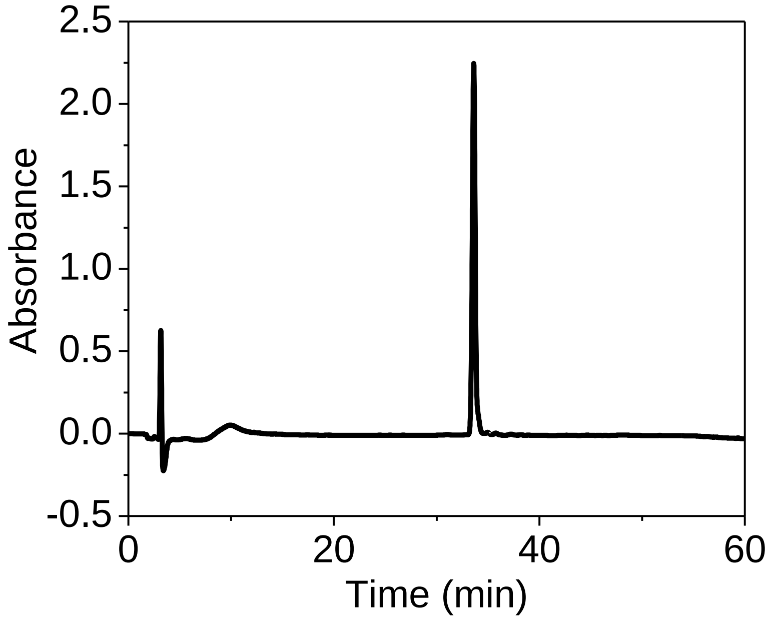


**Figure S2:** Reversed-phase HPLC chromatogram of purified PEP-1.

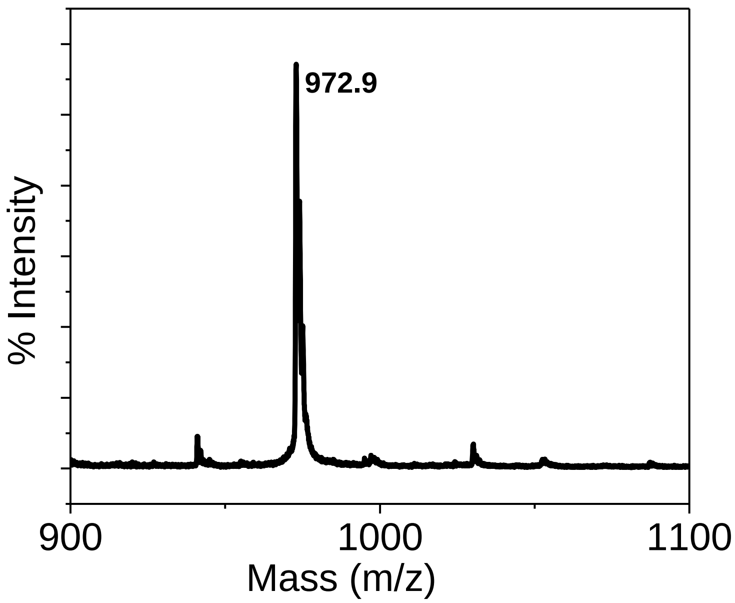


**Figure S3:** MALDI-TOF mass spectrum of purified PEP-1.

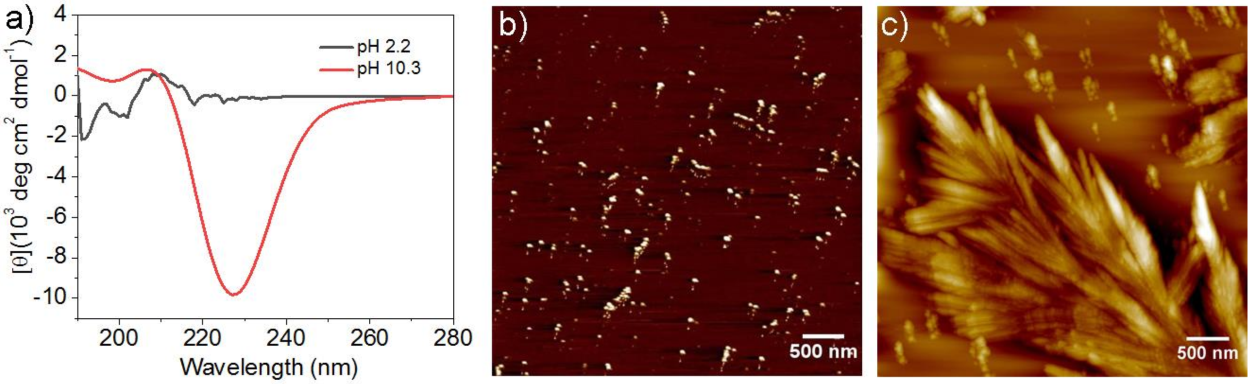


**Figure S4:** a) CD spectra at pH 2.2 and 10.3, respectively. b) AFM images of PEP-1 at pH 2.2 and c) pH 10.3 ($c = 5 \times 10^{-4}$ M, $T = 25$ °C).

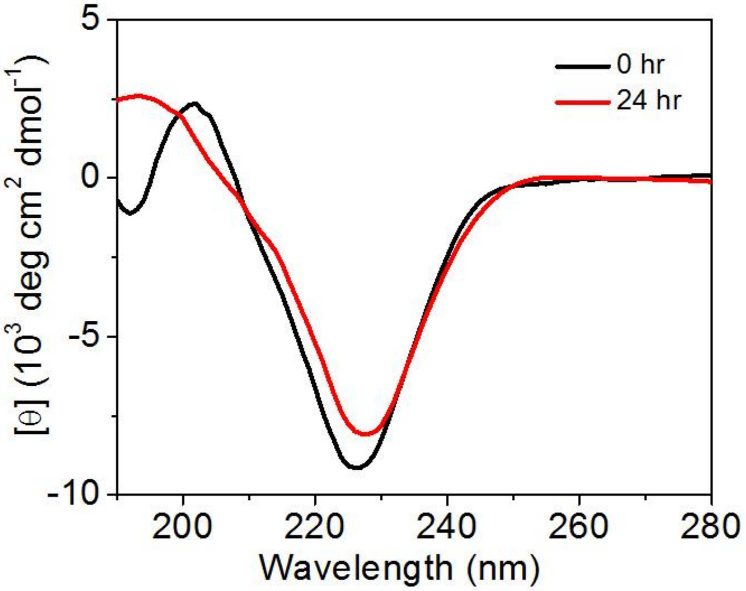


**Figure S5:** Time-dependent CD spectra of PEP-1 at pH 7.4 (PBS buffer; $c = 5 \times 10^{-4}$ M, $T = 25$ °C).

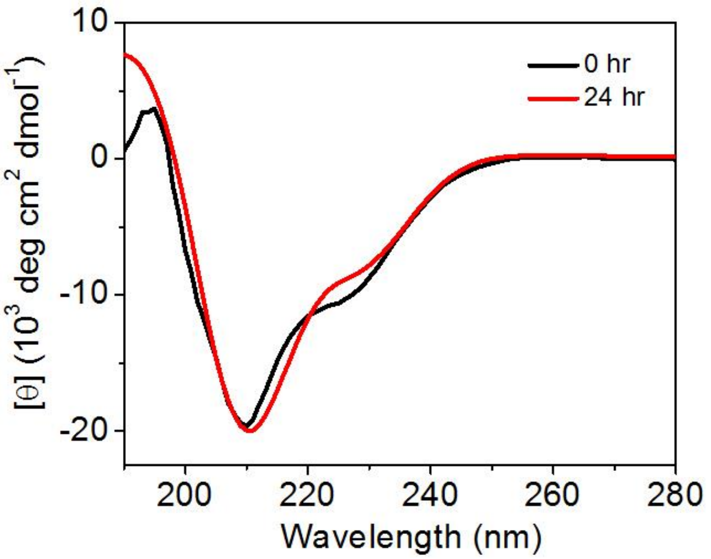


**Figure S6:** Time-dependent CD spectra of PEP-1 at pH 7.4 (PBS buffer; $c = 1.25 \times 10^{-4}$ M, $T$ = 25 °C).

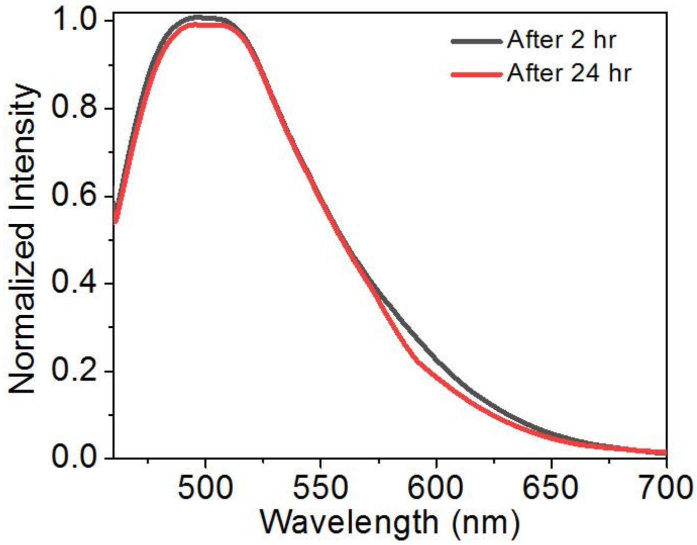


**Figure S7:** Time-dependent ThT assay of PEP-1 at pH 7.4 (PBS buffer; $c = 5 \times 10^{-4}$ M, $T = 25$ °C).

## References

1. Perrin, D.; Armarego, W. L. F.; Perrin, D. R. Purification of Laboratory Chemicals, 2nd ed., *Pergamon, Oxford*, **1980**.
